# Mitochondrial Genome Sequences of the Emerging Fungal Pathogen *Candida auris*

**DOI:** 10.3389/fmicb.2020.560332

**Published:** 2020-10-27

**Authors:** Elizabeth Misas, Nancy A. Chow, Oscar M. Gómez, José F. Muñoz, Juan G. McEwen, Anastasia P. Litvintseva, Oliver K. Clay

**Affiliations:** ^1^Cellular and Molecular Biology Unit, Corporación para Investigaciones Biológicas, Medellín, Colombia; ^2^Wisconsin One Health Consortium, Universidad Nacional de Colombia, Medellín, Colombia; ^3^Mycotic Diseases Branch, Centers for Disease Control and Prevention, Atlanta, GA, United States; ^4^School of Microbiology, Universidad de Antioquia, Medellín, Colombia; ^5^Genoma CES, Universidad CES, Medellín, Colombia; ^6^Broad Institute of MIT and Harvard, Cambridge, MA, United States; ^7^School of Medicine, Universidad de Antioquia, Medellín, Colombia; ^8^Translational Microbiology and Emerging Diseases, School of Medicine and Health Sciences, Universidad del Rosario, Bogotá, Colombia

**Keywords:** mitochondria, comparative genomics, pathogenic fungi, next generation sequencing, annotation

## Abstract

*Candida auris* is an emerging fungal pathogen capable of causing invasive infections in humans. Since its first appearance around 1996, it has been isolated in countries spanning five continents. *C. auris* is a yeast that has the potential to cause outbreaks in hospitals, can survive in adverse conditions, including dry surfaces and high temperatures, and has been frequently misidentified by traditional methods. Furthermore, strains have been identified that are resistant to two and even all three of the main classes of antifungals currently in use. Several nuclear genome assemblies of *C. auris* have been published representing different clades and continents, yet until recently, the mitochondrial genomes (mtDNA chromosomes) of this species and the closely related species of *C. haemulonii, C. duobushaemulonii*, and *C. pseudohaemulonii* had not been analyzed in depth. We used reads from PacBio and Illumina sequencing to obtain a *de novo* reference assembly of the mitochondrial genome of the *C. auris* clade I isolate B8441 from Pakistan. This assembly has a total size of 28.2 kb and contains 13 core protein-coding genes, 25 tRNAs and the 12S and 16S ribosomal subunits. We then performed a comparative analysis by aligning Illumina reads of 129 other isolates from South Asia, Japan, South Africa, and South America with the B8441 reference. The clades of the phylogenetic tree we obtained from the aligned mtDNA sequences were consistent with those derived from the nuclear genome. The mitochondrial genome revealed a generally low genetic variation within clades, although the South Asian clade displayed two sub-branches including strains from both Pakistan and India. In particular, the 86 isolates from Colombia and Venezuela had mtDNA sequences that were all identical at the base level, i.e., a single conserved haplotype or mitochondrial background that exhibited characteristic differences from the Pakistan reference isolate B8441, such as a unique 25-nt insert that may affect function.

## Introduction

*Candida auris* is a yeast capable of causing invasive infections in humans and developing multidrug resistance (Chowdhary et al., 2014). It now has a global distribution spanning five continents (Sharma et al., 2015; Lockhart et al., 2017b; Rhodes et al., 2018; Ruiz-Gaitan et al., 2019). *C. auris* is phylogenetically distant from the most studied pathogenic *Candida* species for humans, such as *C. albicans* and *C. glabrata*, and belongs to a separate clade together with the species *C. haemulonii*, *C. duobushaemulonii*, and *C. pseudohaemulonii*. This clade contains strains that have reduced susceptibility to fluconazole or other azoles, amphotericin B, and/or echinocandins (Cendejas-Bueno et al., 2012). The clinical diagnosis of *C. auris* can be a challenge, and matrix-assisted laser desorption/ionization time of flight (MALDI-TOF) and DNA sequencing are currently the only reliable methods for identification of this pathogen (Caceres et al., 2019).

Only a decade has passed since *C. auris* was first described, following its isolation from the ear canal of a 70-year old Japanese woman in Tokyo (Satoh et al., 2009), and no records of isolates corresponding to this species have been found dating back earlier than 1996 (Kim et al., 2009). Despite the short time since its first emergence, *C. auris* has become a high-priority public health concern in several parts of the world, including South and East Asia, Europe, and North and South America (Lockhart et al., 2017a,b; Escandon et al., 2019). Reasons for the concern include the fungus’ ability to develop multidrug resistance, which can occasionally extend to all of the three main groups of current antifungals (Lockhart et al., 2017a,b; Escandon et al., 2019) and its ability to cause outbreaks in healthcare facilities. Unlike other species of *Candida*, which are primarily associated with the digestive or urogenital systems, *C. auris* can readily colonize patients’ skin and survive on dry abiotic surfaces for weeks contributing to transmission and facilitating outbreaks in healthcare facilities (reviewed in Rossato and Colombo, 2018).

Nuclear genomes have been sequenced for several isolates of *C. auris*, and analysis of the nuclear SNPs from these sequences has allowed four stable clades to be identified, representing different geographical regions, namely South Asia (clade I), East Asia (clade II), South Africa (clade III) and South America (clade IV), with relatively low genetic variation within the clades (Lockhart et al., 2017b); recently a fifth clade was identified, which included a single isolate from Iran (Chow et al., 2019). Progress has been made in sequencing and understanding not only the nuclear genomes of *C. auris* but also those of the other species in its complex, *C. haemulonii*, *C. duobushaemulonii*, and *C. pseudohaemulonii*.

In view of such progress, it is perhaps surprising that full mitochondrial genome (mtDNA, mitogenome) sequences of these pathogens have not been a focus of attention until very recently. There has also been a tendency for fungal mitochondrial genome assemblies to be partly or entirely absent from resources generated by fungal genome projects, perhaps in part because of inherent difficulties in identifying and/or assembling reads in several fungal species (Lynch et al., 2008; Muñoz et al., 2014; Li et al., 2019). Indeed, despite their relatively small size compared to nuclear genomes, mtDNA genomes of fungi and other eukaryotes can contain complex patterns of repeated sequences that not only encumber assembly tasks, but can also lead to ectopic recombination events with subsequent deletion of genome segments, excision of circular subgenomes or other structural genome modifications (Bernardi, 2005a,b; Misas et al., 2016, 2020).

We wished to contribute to bridging the mitochondrial knowledge gap for *C. auris* by first providing an annotated mitochondrial *de novo* sequence assembly of a reference isolate for clade I of *C. auris*, isolate B8441 from Pakistan, and then characterizing variations that are exhibited by strains worldwide using this isolate as reference. Isolate B8441 was chosen as a representative of clade I and because its nuclear genome has previously been sequenced and characterized (Lockhart et al., 2017b). The novel assembly of this isolate complements the recently published first *de novo* genome assembly, including the mtDNA chromosome (Sekizuka et al., 2019), of the original clade II isolate from Tokyo (Satoh et al., 2009), which was obtained independently and in parallel with our clade I mtDNA assembly. We reasoned that the *de novo* assembly, annotation and characterization of the mitochondrial sequence of a clade I isolate together with a comparative analysis would contribute a needed resource and might ultimately provide also a basis for improving our understanding of the fundamental roles played by the mitochondria of *Candida* spp. and other yeasts in controlling cellular networks impacted by antifungal drugs, and in increasing or decreasing virulence or drug susceptibility (Shingu-Vazquez and Traven, 2011). A stand-alone *de novo* assembly has the advantage, over read alignment to an existing assembly of a conspecific strain or congeneric species, that it does not make structural assumptions and is thus less likely to miss insertions, rearrangements or breaks in synteny. Indeed, we found an intron of the *cox1* gene in our clade I mtDNA that is present in clade I but not present in the clade II mtDNA genome assembly of Sekizuka et al. (2019).

After assembling the mtDNA of the reference B8441 and characterizing its protein-coding gene content, tRNAs and genome organization, we performed a comparative analysis on 129 additional *C. auris* isolates from South Asia, Japan, South Africa, and South America. The low genetic variation within clades at the individual base level that we observed among the mitochondrial genomes of *C. auris* agrees with a generally low intra-clade diversity that was previously documented for the nuclear genome of this species (Lockhart et al., 2017a,b). Finally, we focused on the differences between the mitochondrial reference genome and a single haplotype we observed in all 86 isolates from Colombia and Venezuela.

## Materials and Methods

### Sampling and Whole Genome Sequencing Reads

*Candida auris* isolates were collected and DNA extraction and read sequencing were performed previously as part of a larger genome project (Lockhart et al., 2017b; Escandon et al., 2019) that we extend here for the analysis of the mitochondrial genomes.

Read data for all *C. auris* isolates used in the alignments were from the public domain and downloaded from the National Center for Biotechnology Information’s Sequence Read Archive (NCBI/SRA). BioProject IDs included PRJNA328792 for Illumina reads of non-Colombian isolates, PRJNA328792 for the Pac Bio reads of the reference strain B8441 (Lockhart et al., 2017b) and PRJNA470683 for Illumina reads of the Colombian isolates (Escandon et al., 2019).

### *De novo* Assembly and Annotation of the Mitochondrial Genome

The complete genome of isolate B8441 was assembled *de novo* using Canu v-1.5 (Koren et al., 2017) from reads generated as part of a larger genome sequencing project (Lockhart et al., 2017b; Escandon et al., 2019; BioProject ID PRJNA328792). For this Canu assembly we used the following parameters: genomeSize = 24.8m (genome size of *Clavispora lusitaniae*) -pacbio-raw.

The single contig corresponding to the mitochondrial (mtDNA) genome was readily identified on the basis of strong protein-level similarity to the mtDNA gene sets of previously sequenced non-*auris Candida* species, as assessed by tBLASTn (Boratyn et al., 2013) searches against mitochondrial protein sequences of *C. albicans* and *C. lusitaniae*. An apparent nuclear region was removed from the contig, and the ends were joined to circularize the sequence. The resulting sequence was then used as a draft mitochondrial assembly onto which the Illumina reads of the same isolate were mapped using BWA v-0.6.1. The draft sequence was then corrected using Pilon version 1.6 (Walker et al., 2014). Pilon uses read alignment analysis to identify inconsistencies between the input genome and the evidence in the reads. This program can be used for two purposes, to automatically improve draft assemblies and to find variation among strains. We ran Pilon under parameter –fix all to obtain a corrected assembly.

To check the reliability of the entire assembly, we next performed an independent assembly from the same reads using a different assembly tool, and compared the two sequences. We used SPAdes v-3.10 (Bankevich et al., 2012) to assemble *de novo* the complete genome (nuclear and mitochondrial scaffolds) of *C. auris* B8441. SPAdes v-3.10 was run under the –careful mode. This option runs MismatchCorrector, a post-processing tool, which uses the BWA tool to correct the assemblies using the Illumina reads. We obtained assemblies for *k*-mers of length 21, 33, 55, 77, and 99 bp. We selected a *k*-mer size of 99 because it generated the best assembly statistics (Supplementary Table S1). The unique contig corresponding to the mitochondrial genome (mtDNA) was identified in the same way as in the assembly by Canu.

The protein coding genes in the *C. auris* mitogenome were initially predicted by combining output from the programs Glimmer (Delcher et al., 2007), FGENESH (Salamov and Solovyev, 2000), and MITOS (Bernt et al., 2013). The predicted protein coding gene sequences were then translated using NCBI genetic code 4, and identified via BLASTp (Boratyn et al., 2013)^1^ searches against the NCBI non-redundant protein sequence database (nr). The predicted coding sequences (CDS) were then curated by considering alignments with mitochondrial protein coding sequences of other *Candida* species. tRNA genes were predicted with tRNAscan-SE v1.3.1^2^ (Lowe and Eddy, 1997). As a check, we then used Mfannot^3^ (Lang et al., 2007) to obtain an alternative, automatically generated draft annotation of the mitochondrial genome sequence. Agreement was good except for a few inconsistencies, notably in nad genes, in the exact location (± a few nucleotides) of donor/acceptor splice sites (not affecting the final protein sequence), or of coding start sites where several methionine codons followed in close succession. Although in final decisions we typically gave most weight to the reliability of existing annotations of orthologous genes in model organisms such as *Candida albicans*, there may be instances where even trusted reference annotations turn out to be inaccurate.

**FIGURE 1:**
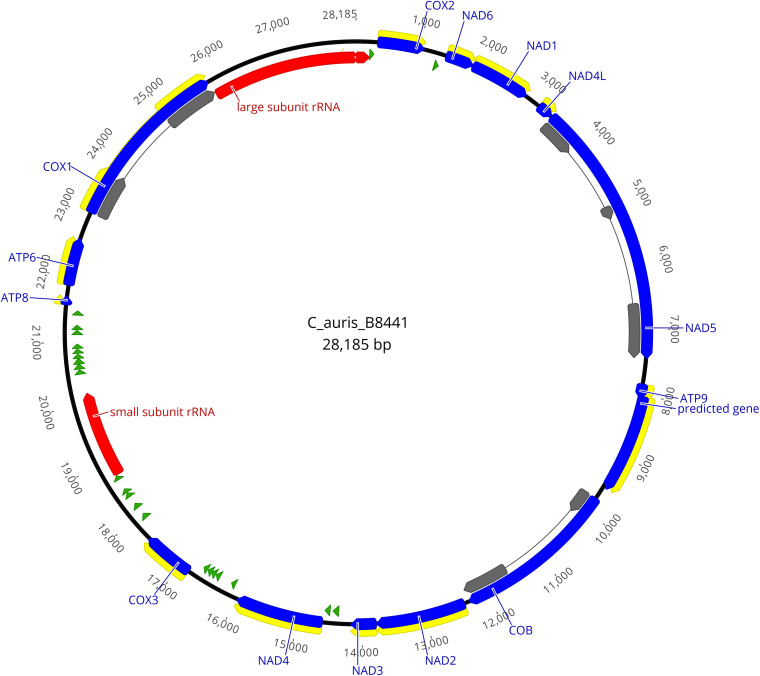
Physical map of the mitochondrial genome of *Candida auris* isolate B8441. Protein coding genes are shown in blue, the exons are shown in yellow or gray, tRNAs are shown in green and rRNAs are shown in red.

The graphical map of the complete mitogenome of isolate B8441 (Figure 1) was drawn with Geneious Pro 11.1.5 (Kearse et al., 2012).

### Phylogenetic Analysis

Our curated assembly of isolate B8441 was used as a reference for single-nucleotide polymorphism (SNP) and other variant calling in read sets from other isolates. SNPs were identified using reference assembly by BWA v–0.5.9 (Li and Durbin, 2009) and variant calling was performed using Pilon v-1.6 under parameter –variant (Walker et al., 2014).

From the SNP calls that had a minimum read depth coverage of 8 (45 SNP positions), which spanned the entire mitochondrial genome, we constructed a phylogenetic tree representing the 130 isolates represented by the read sets, including 81 Colombian, 5 Venezuelan, 10 South African, 17 Pakistani, 16 Indian, and 1 Japanese *C. auris* isolates. The BIC (Bayesian information criterion) score was used to determine the best-fit evolutionary model for the alignment. IQ-tree (Nguyen et al., 2015; Kalyaanamoorthy et al., 2017) was used to construct the phylogenetic tree using Bayesian inference (BI) and Maximum likelihood (ML) methods. Bootstrap values were calculated using 1,000 reiterations.

An additional phylogenetic tree was constructed for 67 isolates of *C. auris*, using BWA to map the Illumina reads to the sequence of the complete mitochondrial genome assembly of reference isolate B8441 (28,212 bp). The resulting sequences were aligned with the ClustalW v-2.1 (Thompson et al., 1994) program and visualized in Geneious Prime v. 2020.1.2 (Kearse et al., 2012), and a maximum likelihood phylogeny was constructed using IQ-Tree v-1.4.4 program (Nguyen et al., 2015) using the HKY nucleotide substitution model and bootstrap analysis based on 1,000 replicates.

## Results

### Assembly and Annotation of *C. auris* Reference Strain B8441

The PacBio RS II sequencing of the total DNA of the *C. auris* reference strain B8441 from Pakistan produced 183,245 reads. The Illumina HiSeq2500 paired-end read sequencing of this isolate produced 7.8 million read pairs with a read length of 251 bp.

Using the Canu assembly program, we obtained an assembly in which the entire mitochondrial genome of B8441 was contained in a single contig (see section “Materials and Methods,” Table 1 and Supplementary Figure S1). This contig had a size of 34.8 kb and contained a nuclear region (∼5.8 kb). The nuclear region matched a region in sequence NW_017263969 of the B8441 nuclear genome assembly reported by Chatterjee et al. (2015; scaffold39, coordinates 34549-2872) and a region in sequence PEKT02000004 of the B8441 nuclear genome assembly reported by Lockhart et al. (2017b; scaffold00008, coordinates 284933-279146). The nuclear region had a higher percentage of GC and lower coverage by Illumina sequencing than the rest of the contig, as expected for a sequence of nuclear origin (Supplementary Figure S2).

**TABLE 1:** Summary of the Canu *de novo* assembly results for *C. auris* isolate B8441.

**Canu assembly stats**	
Sum contig length	12415961
Num contigs	25
Mean contig length	496638
Median contig length	492995
N50 value	759172
Max	1382576

To circularize the sequence of the mitochondrial genome, the mitochondrial contig was aligned against itself using the NUCmer program from the MUMmer v-3.23 package (Kurtz et al., 2004), which allowed the identification of repeated sequences of ∼800 bp at the ends of the mitochondrial contig. The ends were duplicated, indicating the superposition of these sequences as expected in a circular genome (Supplementary Figure S2). We removed the nuclear region and manually joined the ends to obtain a first draft mitochondrial genome assembly, which had a size of 28.2 kb.

To improve base level accuracy of the assembled mtDNA sequence, the Illumina reads of the same isolate were then mapped to it using BWA to obtain the final sequence. The resulting reference assembly showed an average coverage of 4108X. The Pilon program corrected 10 errors, i.e., 2 single nucleotide errors and 8 small indels (Supplementary Table S2).

As a final step of the assembly, we cross-checked the assembly thus obtained by performing an independent *de novo* assembly of the same reads using a different assembly tool. Using the SPAdes assembly program, we obtained an assembly in which the entire mitochondrial genome of B8441 was contained in a single 28.3 kb contig. Its sequence is very similar to the sequence of the assembly by Canu, the only two differences between both sequences are 33 additional bases at the beginning in the contig from Canu and 132 additional bases at the end of the contig from SPAdes.

The PacBio and Canu combination works well to assemble mitochondrial genomes, even those with repetitive regions such as the *Saccharomyces cerevisiae* S288C mitochondrial genome, as reported in the work by Giordano et al. (2017). Possibly, the PacBio library preparation preserves the ratio of mtDNA to nuclear DNA, and the number of mtDNA copies per cell is reflected in greater sequencing depth for mitochondrial sequences than for nuclear sequences (Giordano et al., 2017; Supplementary Figure S2).

Annotation of the corrected mitochondrial assembly was achieved via a combined strategy, using both a homology inference approach that utilized available annotations for *C. lusitaniae* and *C. albicans* and programs for *ab initio* prediction. The resulting annotation was manually re-checked using the R package seqinr (Charif and Lobry, 2007). Where discrepancies were found between methods, the gene annotation was manually curated (see section “Materials and Methods” for details).

The homology-based annotation approach was used to identify the core genes coding for the subunits of the ATP synthase (*ATP6*, *ATP8*, and *ATP9*), the subunits of the reduced nicotinamide adenine dinucleotide ubiquinone oxidoreductase (*NAD1*, *NAD2*, *NAD3*, *NAD4*, *NAD4L*, *NAD5*, and *NAD6*), the subunits of the cytochrome c oxidase enzyme complex (*COX1*, *COX2* and *COX3*), apocytochrome b (*COB*), 23 tRNAs and 2 ribosomal subunits (Figure 1 and Table 2).

**TABLE 2:** Coordinates of genes in the annotation of the mitochondrial genome assembly of isolate B8441 (clade I, Pakistan).

**Gene**	**Function of gene product**	**Strand**	**Coordinates**
			1–28185
*tRNA-Ala*	tRNA	+	220–291
*COX2*	Cytochrome c oxidase	+	335–1066
*tRNA-Asn*	tRNA	+	1273–1343
*NAD6*	NADH dehydrogenase	+	1410–1850
*NAD1*	NADH dehydrogenase	+	1856–2806
*NAD4L*	NADH dehydrogenase	+	3052–3306
*NAD5*	NADH dehydrogenase	+	3306–3887, 4961–5176, 6579–7445
*ATP9*	ATP synthase	+	7845–8075
*COB*	Cytochrome b	+	9748–10140, 11557–12315
*NAD2*	NADH dehydrogenase	+	12381–13784
*NAD3*	NADH dehydrogenase	+	13786–14172
*tRNA-Thr*	tRNA	+	14375–14459
*tRNA-Leu*	tRNA	+	14506–14587
*NAD4*	NADH dehydrogenase	+	14616–15986
*tRNA-Arg*	tRNA	+	16096–16167
*tRNA-Val*	tRNA	+	16379–16450
*tRNA-Trp*	tRNA	+	16453–16523
*tRNA-Phe*	tRNA	+	16530–16600
*tRNA-Lys*	tRNA	+	16608–16679
*COX3*	Cytochrome c oxidase	+	16834–17643
*tRNA-Gln*	tRNA	+	17910–17982
*tRNA-Asp*	tRNA	+	18116–18189
*tRNA-Ser*	tRNA	+	18194–18274
*tRNA-Arg*	tRNA	+	18336–18407
*tRNA-Met*	tRNA	+	18414–18484
*tRNA-Met*	tRNA	+	18652–18722
rns	12S rRNA	+	18738–20152
*tRNA-Ile*	tRNA	+	20478–20548
*tRNA-Leu*	tRNA	+	20555–20635
*tRNA-Tyr*	tRNA	+	20643–20726
*tRNA-His*	tRNA	+	20731–20802
*tRNA-Met*	tRNA	+	20809–20880
*tRNA-Thr*	tRNA	+	20890–20962
*tRNA-Glu*	tRNA	+	21116–21186
*tRNA-Gly*	tRNA	+	21190–21261
*tRNA-Cys*	tRNA	+	21266–21337
*tRNA-Pro*	tRNA	+	21420–21492
*ATP8*	ATP synthase	+	21571–21717
*ATP6*	ATP synthase	+	21880–22620
*COX1*	Cytochrome c oxidase	+	23050–23775, 24919–25806
rnl	16S rRNA	+	25815–28185, 1–219

### Phylogenetic Analysis of the Mitochondrial Genomes of 130 *C. auris* Strains and Within-Clade Variation

Using BWA, we aligned read data from a total of 129 isolates obtained worldwide to the mitochondrial genome of the Pakistan isolate B8441 that we had assembled and annotated. Single-nucleotide polymorphisms/substitutions (SNPs) and other variants such as small insertions/deletions (indels) were identified using Pilon (Supplementary Table S3).

For a phylogenetic analysis using only SNPs, we included 81 Colombian isolates, 5 Venezuelan isolates, 10 South African isolates, 16 from India, 16 additional isolates from Pakistan and one isolate from Japan. Bayesian and maximum likelihood reconstructions gave the same results.

**FIGURE 2:**
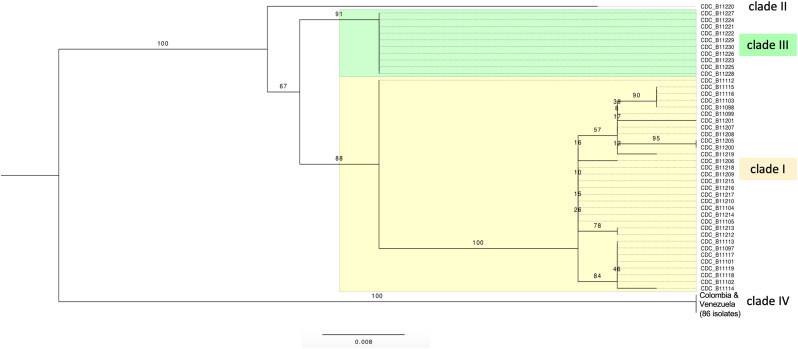
Maximum likelihood tree of *C. auris* strains, obtained using IQ-tree version 1.4.4 (best-fit model TVMe + ASC). The data set included a total of 45 single nucleotide substitution sites for 129 *C. auris* isolates. Bootstrap values calculated using 10,000 reiterations are shown. Four clusters were identified, from top to bottom: South Asia clade (clade I, Pakistan + India, highlighted in yellow), South Africa clade (clade III, highlighted in green), East Asia clade (clade II, Japan, one sequence), and South America clade (clade IV, Colombia + Venezuela, 86 sequences). The rooting is without outgroup and only for display purposes; the branch lengths show the large number of mutations separating the Colombia-Venezuela clade from all other isolates.

Trees based on SNPs from the whole mitochondrial genome showed that isolates were grouped in four clades by geographic region. The isolates of Pakistan and India form the clade I of South Asia and the isolates of Colombia and Venezuela form the clade IV of South America (Figure 2). The same four clades are also observed when the SNPs from whole nuclear genomes are used to construct trees (Lockhart et al., 2017b; Escandon et al., 2019). This concordance shows consistency between nuclear and mitochondrial genomes (Supplementary Figure S3).

Similar to the results for the nuclear genome, very few changes were observed within each clade. The only moderately diverse clade was the South Asian clade I (*n* = 33), where up to 7 SNPs differentiate pairs of isolates. By contrast, the South American (Colombia + Venezuela, *n* = 86) clade IV and South African (*n* = 10) clade III consisted of clonal strains, i.e., no mtDNA differences were exhibited among isolates within the clades.

To confirm the previous results obtained using only SNPs, an additional maximum likelihood tree was constructed, using the sequences of the complete mitochondrial genome assemblies. For 67 isolates of *C. auris* we obtained mitochondrial genome reference assemblies, mapping the Illumina reads to the reference sequence, the mitochondrial genome assembly of isolate *C. auris* B8441 (28,212 bp) we had obtained. This additional analysis gave us the opportunity to include the sequences of seven isolates of Japan that were published more recently by Sekizuka et al. (2019). We again found the four clades described above (Supplementary Figure S4A). In the alignment of the complete mitochondrial genome sequences, which also includes the information of variation resulting from insertion or deletions and not just that from SNPs, we observe a gap of 1143 bp in all eight sequences from Japan, corresponding to the absence of a putative intron in the sequence of the *COX1* gene (Supplementary Figure S4B).

### Differences Between the Clade IV Colombia-Venezuela Mitochondrial Haplotype and the Clade I Pakistan Reference B8441

Since the Colombian and Venezuela isolates of clade IV showed the most striking clonality (Escandon et al., 2019), we focused on the differences between the mitochondrial genome of the Colombia-Venezuela clade and those of clades I, II, and III from outside South America. The mean coverage of the Colombian isolates by Illumina read sets was 4034.06X, and the minimum depth was 124 for the Colombian isolate B11849 (see also Supplementary Table S3). After filtering for quality and read depth, no differences were identified within the clade IV group of 86 Colombian and Venezuelan isolates.

Table 3 shows a summary of the differences we observed between the Colombia-Venezuela haplotype and the Pakistan reference haplotype B8441 that we had assembled *de novo* from long reads and subsequently corrected using high-quality short reads. A full listing of the differences is given in Supplementary Table S4. A total of 25 single nucleotide substitutions and 13 small indels were identified between the clade IV Colombia-Venezuela mitochondrial haplotype and our clade I reference B8441.

**TABLE 3:** Summary of called variants between the reference isolate B8441 (Pakistan, clade I) and the Colombia-Venezuela haplotype (clade IV), using BWA and Pilon.

**Summary Pilon SNP calling**	
Mapped reads range	(142253–979042)
Mean coverage range	(1244–7797)
SNPs (single nucleotide substitutions)	25
Small insertion	7 (total 33 bases)
Small deletions	6 (total 10 bases)

Of the 38 variants differentiating genomes of the clade IV Colombian/Venezuelan isolates from the B8441 clade I reference genome from Pakistan, only two were located in the protein-coding region of a core gene, namely in *NAD5* and in *ATP6*, and neither of those two changes altered the protein sequence (Table 3 and Supplementary Table S4).

**FIGURE 3:**
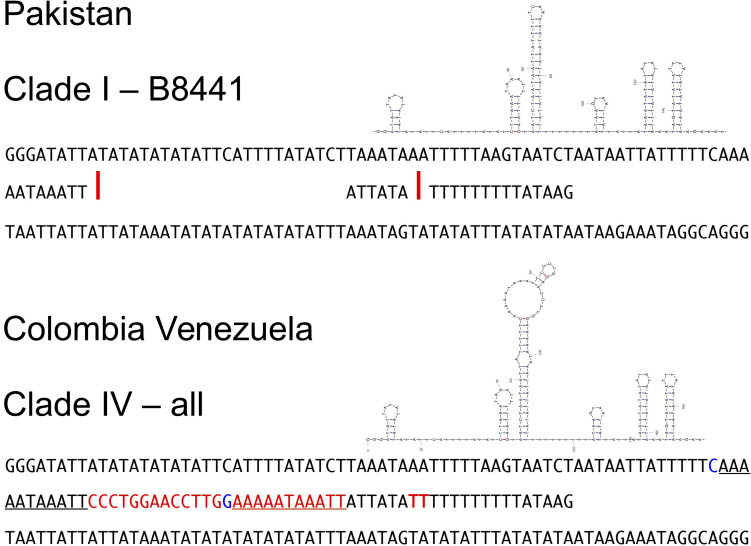
Comparison of a segment of the mtDNA genomic sequence surrounding the longest insert (25 + 2 nt, red letters) observed in the clade IV haplotype from Colombia and Venezuela (bottom), in the intergenic region between *NAD5* and *tRNA*-Ser, and the corresponding sequence of reference clade I isolate B8441 from Pakistan (top). Stemloop diagrams show one of the predicted optimal structures of the clade IV sequence, obtained using UNAFold with default settings (Markham and Zuker, 2008), which has a more complex stemloop component that is absent among the simpler optimal structures predicted for the B8441 sequence. A subsequence that is repeated in the clade IV haplotype but not in the B8441 isolate is underlined. A possible C–G pairing that could thermodynamically lead to the different folding shown for the clade IV haplotype is shown in blue letters. Red letters and bars indicate, respectively, the bases of the clade IV haplotype that are absent in the clade I haplotype, and the corresponding positions in the clade I haplotype (B8441 mtDNA genome coordinates 14,285 and 14,291).

The Colombia-Venezuela haplotype differs from the reference B8441 by more than a few nucleotides in the intergenic region between *NAD5* and *tRNA*-Ser, where there is a 25-nt insert containing a locally repeated nucleotide motif, and then shortly following it, another insert of 2 nt in a poly-T stretch (Figure 3). Examination of the single reads at the location using blastn_vdb confirmed that that the 25-nt insert was not merely an assembly/alignment artifact, nor a result of a heteroplasmy in which only some of the mitochondrial genome copies contain the insert. Indeed, at this location numerous reads (>600 in read set SRR7140045) perfectly matched the Colombia-Venezuela haplotype, while no reads corresponded to the insert-free B8441 haplotype.

The repetitive nature of the resulting sequence prompted us to look at likely secondary structures of the region in both the Colombian-Venezuela and Pakistan B8441 haplotypes, using UNAfold. We found a corresponding predicted structure uniquely in the Colombia-Venezuela haplotype, having a longer and bulkier stem-loop, which was not present in the optimal structures predicted for the insert-free B8441 haplotype (Figure 3).

### The *de novo* Assembly of Clade I Contains an Intron in the *COX1* Gene That Is Not Present in Clade II Sequences

Apart from the inter-clade differences between clades I and IV presented above, we also noted that clade I and clade II exhibited a clear structural difference in the *COX1* gene, where an intron that is present in our clade I sequence was absent not only in the Japanese isolate assembled from clade II (Sekizuka et al., 2019), but also in seven other Japanese strains from clade II for which reads were available (Supplementary Figure S4B). Although it may be too early to conclude that this feature is characteristic of all of clade II until more of this clade’s isolates are sequenced, it is clear that the missing *COX1* intron feature distinguishes at least a part of clade II from the other isolates worldwide that have been sequenced so far (Supplementary Figure S4). As a methodological note, we point out that an automated reference (instead of *de novo*) assembly of clade I, using the existing clade II sequence as the reference, might have missed this structural difference, and would not have been able to generate the sequence of the intron.

### Complexity Within the South Asian Clade I

As mentioned above, the only clade, among the four clades we analyzed, that exhibited some degree of intra-clade complexity was the South Asian clade (clade I; tree in Figure 2 and Supplementary Figure S4A), which contained sub-branches that did not simply correspond to the geographic distribution within South Asia. Although the number of isolates in these inner branches within clade I represented by read sets was low, we mapped antifungal susceptibility data from CDC onto the tree, and found that only one partition, of 10 strains into two small subtrees of sizes 3 and 7, suggested a possible association between mtDNA variation and presence/absence of resistance to amphotericin B (*p* = 0.033 for a nominal Fisher’s exact test) or the corresponding log MIC values (*p* = 0.0037 for a nominal *t-*test). Each of the two groups contained isolates from both Pakistan (P) and India (I). The susceptible group (CBC-B11 isolates, with country of origin and MIC values: 099/P/1, 207/I/0.75, 208/I/0.75) was characterized by the consensus motif, GGAAA, of the three haplotype motifs of the contrasting group, consisting of 6 resistant and 1 borderline isolates. The three haplotypes were AGAAA (098/P/1.5, 103/P/1, 115/P/2, 116P//1.5), GGATT (200/I/1.5, 205/I/4) and GTCAA (201/I/2). The five SNPs of the motifs span 3 different genomic locations, namely (from left to right) position 5,365 between exon 2 and exon 3 of *NAD5*, positions 18,319–18,320 between *tRNA-*Ser and *tRNA-*Arg, and positions 19,481–19,482 in the first exon of the 12S rDNA gene *rns*. Since these mutations are in mtDNA their presence may or may not suggest a causal role in resistance (see section “Discussion”). If they, and the MIC value ranges of their carriers, turn out to be supported by larger sample sizes in future, then a basic hypothesis to consider would be that of a non-resistant strain (haplotype with motif GGAAA) that acquired AmB resistance on three different occasions. For now, we just flag this part of the clade I for possible future interest.

## Discussion

In this work we assembled *de novo* and annotated the mitochondrial genome of the B8441 isolate of *C. auris*, which represents a pathogenic South Asian clade (clade I) of this species.

Recently, the first annotated assembly of a mitochondrial genome of *C. auris* was released and published, for an isolate from Japan belonging to the East Asian clade II (isolate JCM 15448, or B11220 in the numbering we have used here, with sequence accession number AP018713; 2019-10-11) (Sekizuka et al., 2019). This recent sequence gave us an opportunity to double check our finished sequence of the clade I isolate B8441 for base-level accuracy, after discounting the 11 single-nucleotide differences and the *COX1* intron’s presence/absence difference, compared to B8441, that we had observed when aligning the reads of the JCM 15448 isolate to our B8441 assembly (Supplementary Table S3). We found perfect agreement with the sequence reported in Sekizuka et al. (2019) at the individual-base level.

In the article of Sekizuka et al. (2019), the authors compared their sequence with six other mtDNA haplotypes, as well as with 126 additional *C. auris* strains deposited in the SRA database, and with *a posteriori* annotated mtDNA sequences of the previously sequenced *C. duobushaemulonii* and *C. haemulonii* isolates, thus providing a comparative analysis extending beyond *C. auris*. As the authors mentioned, the Japanese strains might differ from those isolated in other countries, which are often resistant to one or more antifungal drugs and are highly pathogenic, so the present work complements well the work of Sekizuka et al. (2019).

For the larger species complex that includes not just *C. auris* but also *C. haemulonii, C. duobushaemulonii* and *C. pseudohaemulonii*, unannotated mitochondrial contigs for an amphotericin B-resistant strain of *C. duobushaemulonii* (isolate B09383, sequence PKFP01000007) and for a multi-drug resistant strain of *C. haemulonii* (isolate B11899, sequence PKFO01000012) had been previously obtained as part of a whole genome sequencing project (Munoz et al., 2018).

**FIGURE 4:**
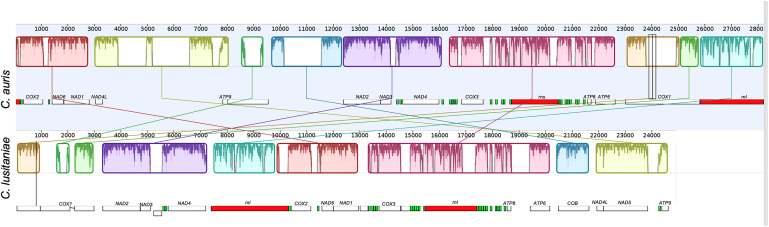
Alignment of the complete mitochondrial genomes of *C. auris* and *Clavispora lusitaniae*, obtained using Mauve (Darling et al., 2010). The mitochondrial genome assembly of the clade I isolate B8441 from Pakistan, sequenced on PacBio RS II (top), and the genome assembly of *Clavispora lusitaniae* KC993186 (bottom) are shown. The colored big boxes represent Locally Collinear Blocks (LCBs), i.e., conserved segments that appear to be internally free from genome rearrangements. The order of the LCBs is given by the original coordinates of the sequences specified by the assembly program and the coordinates in the previously reported sequences when they are presented as a linear sequence. The plot inside the boxes represents the local quality of the alignment. The small boxes represent the genes, protein coding genes are shown in white, ribosomal genes and tRNAs are shown in red and green respectively.

In comparison with other mitogenomes of the *Candida* genus, the mitochondrial genome of *C. auris* is larger than that of *C. lusitaniae* (28.2 vs. 24.8 kb; Figure 4), although the content of protein coding genes is the same.

Recent advances in high-throughput sequencing technologies now present new opportunities to explore the evolution of the mitochondrial (mtDNA) genome. Although compared to metazoans, studies on the mitochondrial genomes of fungal species are limited (Aguileta et al., 2014), Saccharomycotina yeasts have proven to be the leading organisms chosen for comparative and population genomics of mitochondrial sequences (Freel et al., 2015). As a result, there are already a considerable number of mitochondrial sequences available for this group of fungi. An objective of the present study was to generate a *de novo* assembly and contribute a carefully curated annotation of a mitochondrial genome representing a pathogenic clade (South Asia, clade I) of *C. auris*. We then used reference assemblies including other read sets of isolates from around the world, which we hope will facilitate the inclusion of this pathogen in future comparative genomic work.

Further study of the mitochondrial genomes of pathogenic fungi could help to identify new target proteins with therapeutic potential, as even the conserved mitochondrial proteins, which are homologous in fungi and humans, differ in their levels of affinity for the same inhibitor or drug (Vincent et al., 2016; Jeffery-Smith et al., 2018). There is also the question of how the understanding of drug resistance and its possible tradeoffs for the fungus (Vincent et al., 2013) could benefit from deeper knowledge of previously identified roles of mitochondria and their genes and genomes.

In the case of *C. auris*, several studies have demonstrated that some clinical isolates of *C. auris* show a high tolerance to the antifungals amphotericin B (AmB) and fluconazole (Kathuria et al., 2015). Nuclear genes having mutations reported as associated with resistance to AmB in *Candida* spp. include various genes of the ergosterol biosynthesis pathway (Sanglard, 2019). More recently in *C. auris* the gene for Flo8 has been added to the list (Escandon et al., 2019; Misas et al., 2019; Sanglard, 2019), which has roles in filamentation and the production of pseudohyphae in other yeasts (Liu et al., 1996; Misas et al., 2019). Encumbered filamentation is one of the correlates of AmB resistance observed in experiments on *C. albicans*, as is hypersensitivity to oxidative stress (Vincent et al., 2013), where mitochondrial function is clearly relevant.

Apart from tradeoffs or side-effects of mutations that help confer resistance, there is for AmB also the question of how the fungus accommodates to situations where it has to live with drastically reduced levels of ergosterol, the target of AmB. In such situations, mitochondria can play a key role, as they can critically affect and can be critically affected by changes in ergosterol abundance (Cirigliano et al., 2019). Furthermore, since a conserved retrograde pathway exists in fungi (Butow and Avadhani, 2004; Liu and Butow, 2006) that can be triggered in an attempt to mitigate consequences of a defective mitochondrial response, the outcomes of ergosterol deprivation for the cell and its mitochondria may depend on the conditions encountered, as has been studied in *S. cerevisiae* (Cirigliano et al., 2019). A series of studies of loss of mitochondrial function in azole resistant isolates of *Candida glabrata* are described in Ferrari et al. (2011a, b) and Vale-Silva and Sanglard (2015).

Mitochondrial mutations that are found in drug resistant strains could thus exist either as part of a resistance strategy (i.e., be causally involved in creating or maintaining resistance) or, where AmB is the drug, be instead simply a marker of a general neglect of repair, control or maintenance of the mitochondrial genome, resulting from ergosterol shortage and leading to no gain of function. The latter type of mutation could in principle occur in surviving susceptible, or partly resistant, strains under AmB assault. Mutations in mtDNA, whether at the SNP level or structural, might then be of two types: those that contribute to a cause of resistance and could inform on its causal molecular mechanisms (as in genes of the nuclear genome), and those merely representing consequences of ergosterol deficiency impacting on mtDNA maintenance, which could in principle occur in various parts of the mitochondrial genome and might provide no specific information.

Our read sequencing and comparative analysis of a large number of *C. auris* strains from different geographic origins, and in particular our comparison of the clade I Pakistan reference isolate B8441 with the clade IV isolates from Colombia and Venezuela, revealed a very low mitochondrial sequence diversity among strains of the latter clade, in line with observations at the nuclear genome level (Lockhart et al., 2017b). Thus, although the trees we obtained were consistent with expected groupings, the sequence comparisons suggest that mitochondrial genomes of *C. auris* are typically less likely to be useful for *de novo* discovery of phylogenetic relationships (or for subtyping) within this species’ main clades because of the low number of differences. As an extreme example, the 81 Colombian isolates and 5 Venezuela isolates had identical mtDNA sequences at the base level. Such paucity of mtDNA genomic diversity can limit the ability of mtDNA mutation screening to inform on potentially associated phenotypic traits or tendencies such as resistance or virulence. Indeed, for susceptibility and mtDNA data available to us, we identified only one small region, within clade I, where such associations might be supported.

Constancy such as is observed for the mitochondrial haplotype of Colombia and Venezuela would, however, provide an invariant genetic background against which nuclear variation could be viewed.

For example, a recent study of the outbreaks that occurred in Colombia (South America) during 2015–2016, comparing whole genome nuclear genome assemblies of the same 81 isolates from 4 Colombian hospitals that we study here in an mtDNA context, led to the conclusion that transmission of *C. auris* had occurred both within and between hospitals at the regional level. Furthermore, a regional increase in amphotericin B (AmB) resistance was seen to correspond to a subclade of the Colombian isolates as determined from the nuclear genomes. This subclade is distinguished by a small number of non-synonymous point substitutions, including a substitution in the gene for a well-studied transcription factor, Flo8, that is present in many fungi (Escandon et al., 2019; Misas et al., 2019). Apart from a role in filamentation mentioned above, a number of other functional properties and structural features of Flo8 render it possible that the Flo8 mutation as observed in *C. auris* could correspond to either potentiation and/or loss of function, depending on the context (Misas et al., 2019).

The conserved mtDNA genome of the Colombian and Venezuelan (clade IV) isolates of *C. auris* showed characteristic differences when compared with the Pakistan (clade I) isolates. The largest single difference we observed is a 25-nt indel that could in principle change the local secondary structure enough to have functional consequences (Figure 3). It is conceivable that the more complex structure of the Colombian/Venezuelan haplotype might, for example, offer a target for a protein such as a single strand binding protein, or influence replication efficiency as some *ori* loops, which can be sensitively dependent on base level changes, do in mtDNA genomes of *S. cerevisiae*; reviewed in Bernardi (2005a, b).

It would also be conceivable that the landscape of simple stemloops in the reference haplotype of B8441 might raise the probability of ectopic intrachromosomal recombination events leading to large-scale structural mtDNA variants (see, e.g., Van Dyck et al., 1992; Reddy et al., 2000), Figures 3, 4 of Damas et al. (2012) and Misas et al. (2016). Secondary structures at different scales that can be formed by fungal mtDNA genomes, which can affect replication, transcription, and replication in *S. cerevisiae* (Bernardi, 2005a,b) have to our knowledge not yet been investigated in *C. auris* or its close relatives.

For example, the clade I segment might be more prone than the clade IV segment to undergo a recombination event with another segment elsewhere in the mtDNA genome in which intervening DNA is deleted, and such a difference in mtDNA genome integrity might in turn affect virulence or resistance (Shingu-Vazquez and Traven, 2011; Vale-Silva and Sanglard, 2015). More generally, the benefit of nuclear changes for the fungus could depend on the integrity, heteroplasmy level or functioning of the mitochondrial genome, which could in turn be influenced by some or more of the mtDNA sequence differences reported here.

## Conclusion

We have provided the first *de novo* assembled and annotated sequence of the mitochondrial (mtDNA) genome of clade I of the newly emerging yeast *C. auris*. Since several studies have demonstrated that mitochondrial function can modulate the virulence and antifungal resistance in fungi of the *Candida* genus, the sequence of the mitochondrial genome assembly could contribute to elucidating the participation of the mitochondria in these mechanisms in *C. auris*. Studies of the mitochondrial genome of pathogenic fungi might also help to identify new target proteins with therapeutic potential, which could be of much value as some isolates are resistant to all three conventional anti-fungal treatment lines (azoles, amphotericin B, and echinocandins).

## Data Availability Statement

The datasets generated or analyzed in this study can be found in NCBI under project accession numbers PRJNA328792 and PRJNA470683. The annotation of the mitochondrial genome assembly of isolate B8441 generated in this study, GenBank accession number MT849287, is also given in Table 2 of the main text.

## Author Contributions

EM, JGM, and OC conceptualized and designed the study. EM assembled, annotated, and analyzed the data of *C*. *auris* mitochondrial genomes. NC and AL provided sequences and guidance. NC, OG, and JFM contributed in bioinformatic analyses. All authors wrote the manuscript.

## Disclaimer

The use of product names in this manuscript does not imply their endorsement by the U.S. Department of Health and Human Services. The finding and conclusions in this article are those of the authors and do not necessarily represent the views of the Centers for Disease Control and Prevention.

## Conflict of Interest

The authors declare that the research was conducted in the absence of any commercial or financial relationships that could be construed as a potential conflict of interest. The reviewer RF declared past co-authorship with several of the authors, AL, JFM, and NC, to the handling editor.

## Footnotes

1 https://blast.ncbi.nlm.nih.gov/Blast.cgi?PAGE=Proteins

2 http://lowelab.ucsc.edu/tRNAscan-SE/

3 https://megasun.bch.umontreal.ca/RNAweasel/
