# Supplementary material for: Mitochondrial Genome Sequences of the Emerging Fungal Pathogen Candida auris
Source: Front Microbiol. 2020 Oct 27;11:560332. doi: 10.3389/fmicb.2020.560332 (PMC7652928; doi:10.3389/fmicb.2020.560332)
Supplement: Supplementary file 1 [file Presentation_1.PDF]

Supplementary Material

Legend to Supplementary Table S3

Supplementary Figures S1 – S4

Legend to Supplementary Table S3 (in separate Excel file): Accession numbers, geographical origins, BWA results and SNP calling results for the 129 additional sequenced mitochondrial genomes of *C. auris* are shown in "Information for 130 isolates" (worksheet 1). Since the Colombian and Venezuelan isolates all had identical SNP variants, in "All SNVs in 130 isolates" (worksheet 2) and "FixBreak diagnostics" (worksheet 3) only those for the 5 Venezuelan strains are shown.

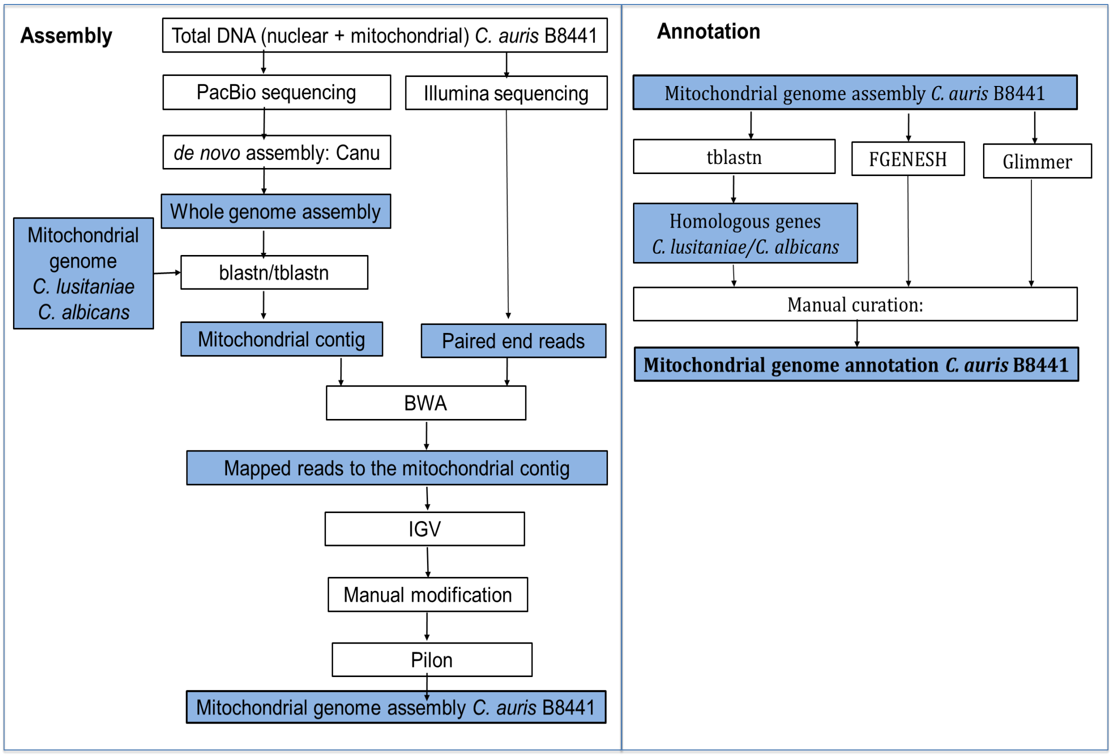

Supplementary Figure S1: Workflow of mitochondrial genome assembly and annotation.

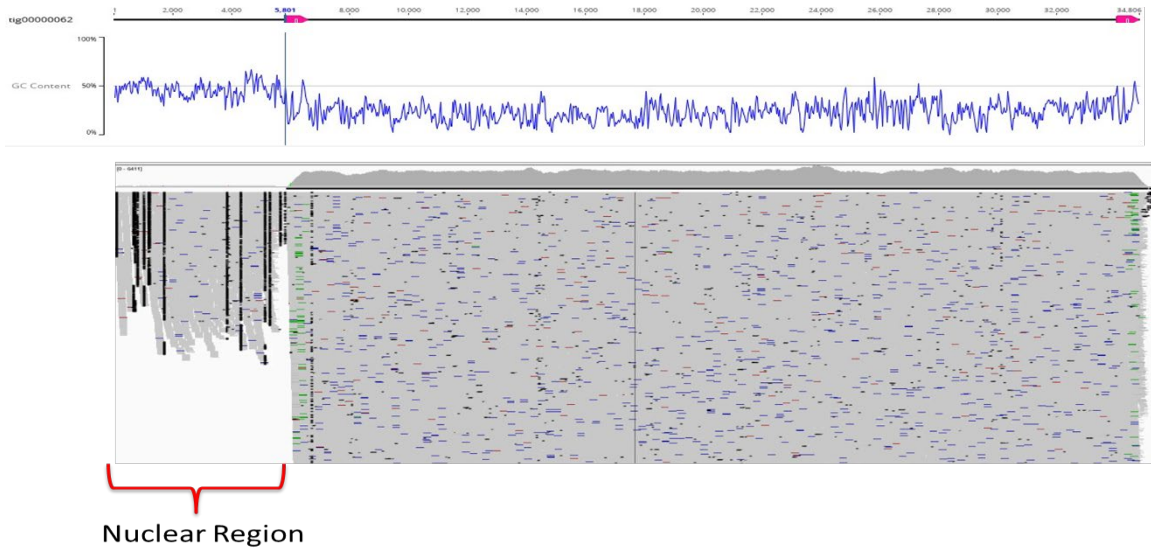

Supplementary Figure S2: GC moving-window plot (blue track) and repeat regions (two pink boxes) of the complete mitochondrial genome of *C. auris* isolate B8441, drawn using Geneious Pro v-11.1.5, and (bottom, gray track) Illumina mapped reads image from IGV.

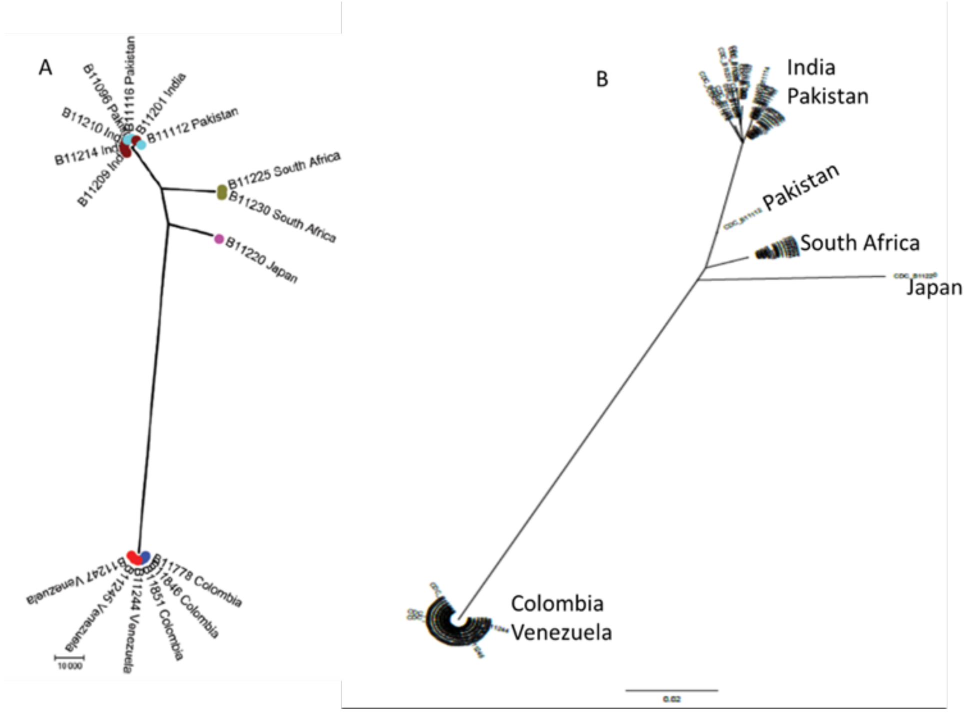

Supplementary Figure S3: Comparison of the phylogenetic reconstruction from the SNPs of the complete nuclear genome (A), from Escandon et al. (2019), and the phylogenetic reconstruction from the SNPs of the complete mitochondrial genome (B), showing that the two topologies are very similar, and both with very long branches leading to the Colombia-Venezuela clade (clade IV).

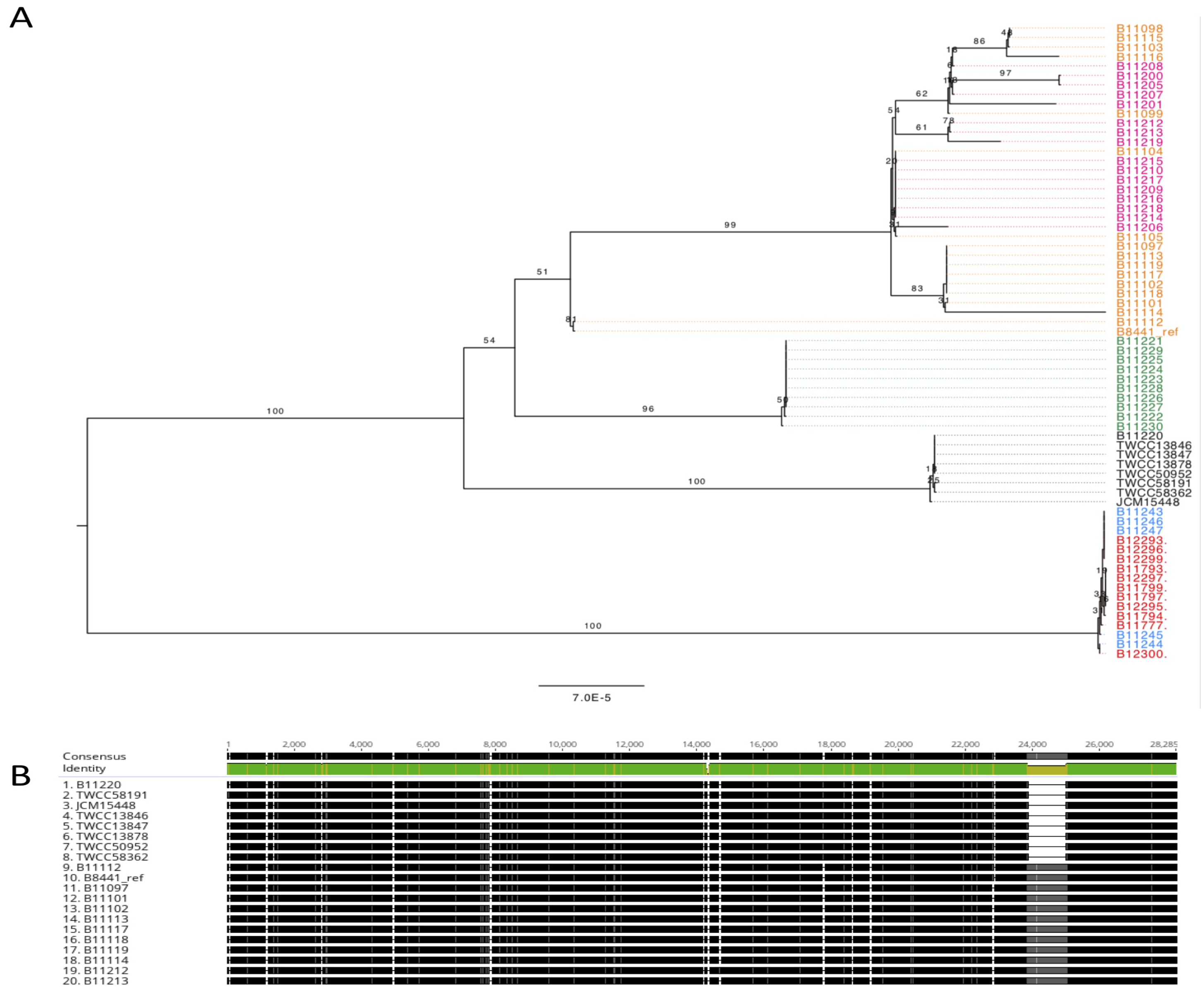
A
B11098
B11115
B11103
B11116
B11208
B11200
B11205
B11207
B11201
B11099
B11212
B11213
B11219
B11104
B11215
B11210
B11217
B11209
B11216
B11218
B11214
B11206
B11105
B11097
B11113
B11119
B11117
B11102
B11118
B11101
B11114
B11112
B8441_ref
B11221
B11229
B11225
B11224
B11223
B11228
B11226
B11227
B11222
B11230
B11220
TWCC13846
TWCC13847
TWCC13878
TWCC50952
TWCC58191
TWCC58362
JCM15448
B11243
B11246
B11247
B12293.
B12296.
B12299.
B11793.
B12297.
B11799.
B11797.
B12295.
B11794.
B11777.
B11245
B11244
B12300.
7.0E-5
B
Consensus
Identity
1. B11220
2. TWCC58191
3. JCM15448
4. TWCC13846
5. TWCC13847
6. TWCC13878
7. TWCC50952
8. TWCC58362
9. B11112
10. B8441_ref
11. B11097
12. B11101
13. B11102
14. B11113
15. B11117
16. B11118
17. B11119
18. B11114
19. B11212
20. B11213
1
2,000
4,000
6,000
8,000
10,000
12,000
14,000
16,000
18,000
20,000
22,000
24,000
26,000
28,285

Supplementary Figure S4: (A) Maximum likelihood tree *of C. auris* strains, obtained using IQ-tree version 1.4.4 (best-fit model HKY). The alignment length was 28,285 bp for 67 *C. auris* isolates, bootstrap values calculated using 1000 reiterations are shown. Four previously identified clusters were confirmed, from top to bottom: South Asia clade (clade I), represented by 17 isolates from Pakistan, shown in orange and 16 isolates from India, shown in pink; South Africa clade (clade III), represented by 10 isolates, shown in green; Japan clade (clade II), including 8 isolates, of which 7 were recently published by Sekizuka et. al. (2019), shown in black; and South America clade (clade IV), represented by 11 isolates from Colombia, shown in red and 5 isolates from Venezuela, shown in blue. (B). Region of the alignment of the mitochondrial genome sequences of 8 isolates from Japan and 12 isolates belonging to the South Asia cluster, showing the 1143 bp gap present in the sequences from Japan. The sequences were aligned with ClustalW v-2.1 and visualized in Geneious Prime version 2020.1.2.
