# Supplementary material for: Mitochondrial Genome Sequences of the Emerging Fungal Pathogen Candida auris
Source: Front Microbiol. 2020 Oct 27;11:560332. doi: 10.3389/fmicb.2020.560332 (PMC7652928; doi:10.3389/fmicb.2020.560332)
Supplement: Supplementary file 2 [file Table_1.DOCX]

**Supplementary Table S1:** Summary of the SPAdes *de novo* assemblies results for *C. auris* isolate B8441

|  | ***k* = 21** | ***k* = 33** | ***k* = 55** | ***k* = 77** | ***k* = 99** |
| --- | --- | --- | --- | --- | --- |
| Percentage N | 0 | 0 | 0 | 0 | 0 |
| Sum contig length (bp) | 13320322 | 13244950 | 13212469 | 13150914 | 13090351 |
| Number of contigs | 13227 | 5265 | 3252 | 2453 | 1869 |
| Mean contig length (bp) | 1007 | 2515 | 4062 | 5361 | 7003 |
| Median contig length (bp) | 35 | 336 | 394 | 420 | 441 |
| N50 value (bp) | 6056 | 19040 | 36598 | 48051 | 78920 |
