# Supplementary material for: Mitochondrial Genome Sequences of the Emerging Fungal Pathogen Candida auris
Source: Front Microbiol. 2020 Oct 27;11:560332. doi: 10.3389/fmicb.2020.560332 (PMC7652928; doi:10.3389/fmicb.2020.560332)
Supplement: Supplementary file 3 [file Table_2.DOCX]

**Supplementary Table S2:** Summary of changes introduced by Pilon to correct false nucleotides and artifactual indels in the initial PacBio assembly

| Summary of Pilon corrections | |
| --- | --- |
| Mapped reads | 514530 |
| Mean coverage | 4108X |
| Single nucleotide replacements | 2 |
| Small insertions | 6 (total 8 bases) |
| Small deletions | 2 (total 2 bases) |
