# Supplementary material for: Mitochondrial Genome Sequences of the Emerging Fungal Pathogen Candida auris
Source: Front Microbiol. 2020 Oct 27;11:560332. doi: 10.3389/fmicb.2020.560332 (PMC7652928; doi:10.3389/fmicb.2020.560332)
Supplement: Supplementary file 5 [file Table_4.DOCX]

**Supplementary Table S4:** List of differences between clade IV Colombia-Venezuela mitochondrial haplotype and the clade I Pakistan isolate B8441

| Position in ref. sequence (B8441) | Type of change | REF | ALT | Upstream gene | Distance to closest upstream gene | Downstream gene | Distance to closest downstream gene |
| --- | --- | --- | --- | --- | --- | --- | --- |
| 1166 | del | CC | . | cox2 | 831 | tRNA-Asn | 107 |
| 1168 | snp | C | T | cox2 | 833 | tRNA-Asn | 105 |
| 1386 | del | A | . | tRNA-Asn | 113 | nad6 | 24 |
| 1517 | snp | A | G | nad6 | 107 | nad1 | 333 |
| 2638 | snp | T | C | nad1 | 788 | nad4l | 414 |
| 2820 | del | TTT | . | nad1 | 970 | nad4l | 232 |
| 2824 | snp | T | A | nad1 | 974 | nad4l | 228 |
| 2852 | snp | T | A | nad1 | 1002 | nad4l | 200 |
| 2929 | snp | T | A | nad1 | 1079 | nad4l | 123 |
| 2945 | snp | T | A | nad1 | 1095 | nad4l | 107 |
| 2983 | snp | G | A | nad1 | 1133 | nad4l | 69 |
| 4313 | snp | T | C | nad5 intron1 | 0 |  |  |
| 4936 | ins | . | A | nad5 intron1 | 0 |  |  |
| 5700 | snp | T | A | nad5 intron2 | 0 |  |  |
| 6821 | snp | G | A | nad5 exon3 | 0 |  |  |
| 7542 | del | TA | . | nad5 | 4236 | atp9 | 303 |
| 7717 | snp | G | T | nad5 | 4411 | atp9 | 128 |
| 7759 | del | TA | . | nad5 | 4453 | atp9 | 86 |
| 7784 | ins | . | G | nad5 | 4478 | atp9 | 61 |
| 7788 | snp | A | G | nad5 | 4482 | atp9 | 57 |
| 7830 | del | A | . | nad5 | 4524 | atp9 | 15 |
| 8630 | snp | C | T | atp9 | 785 | cob | 1118 |
| 8654 | snp | G | A | atp9 | 809 | cob | 1094 |
| 9580 | snp | C | T | atp9 | 1735 | cob | 168 |
| 10326 | snp | G | A | cob intron | 0 |  |  |
| 14274 | ins | . | AAAAATAAATTCCCTGGAACCTTGG | nad3 | 488 | tRNA-Ser | 101 |
| 14291 | ins | . | TT | nad3 | 505 | tRNA-Ser | 84 |
| 15605 | snp | C | T | nad4 | 989 | tRNA-Arg | 491 |
| 18562 | snp | C | A | tRNA-Met 1 | 148 | tRNA-Met 2 | 90 |
| 18565 | ins | . | GG | tRNA-Met 1 | 151 | tRNA-Met 2 | 87 |
| 18602 | snp | A | C | tRNA-Met 1 | 188 | tRNA-Met 2 | 50 |
| 19085 | ins | . | G | rns | 0 |  |  |
| 21876 | snp | T | A | atp8 | 305 | atp6 | 4 |
| 22266 | snp | A | T | atp6 | 0 |  |  |
| 22757 | ins | . | A | atp6 | 877 | cox1 | 293 |
| 22758 | snp | T | A | atp6 | 878 | cox1 | 293 |
| 22759 | snp | G | A | atp6 | 879 | cox1 | 291 |
| 24042 | snp | C | T | cox1 intron | 0 |  |  |
